# Antimicrobial Efficacy of Photodynamic Therapy as an Adjunct to Brix 3000 in Minimally Invasive Management of Carious Lesions in Primary Teeth

**DOI:** 10.3390/ph19020310

**Published:** 2026-02-12

**Authors:** Zornitsa Lazarova, Raina Gergova, Nadezhda Mitova

**Affiliations:** 1 Department of Pediatric Dentistry, Faculty of Dental Medicine, Medical University—Sofia, 1 Georgi Sofiyski St., 1431 Sofia, Bulgaria; n.mitova@fdm.mu-sofia.bg; 2 Department of Medical Microbiology, Medical Faculty, Medical University—Sofia, 2 “Zdrave” Str., 1431 Sofia, Bulgaria; r.gergova@medfac.mu-sofia.bg

**Keywords:** photodynamic therapy, photosensitizer, LED activation, Brix 3000, minimally invasive dentistry, antimicrobial disinfection, primary teeth, *Streptococcus mutans*

## Abstract

**Background/Objectives**: In the context of minimally invasive dentistry, photodynamic therapy (PDT) is regarded as a biologically oriented method for controlling microbial activity during caries excavation. Brix 3000 is a modern material used in chemo-mechanical removal of carious lesions, enabling selective elimination of infected dentin. The present study compares the antimicrobial effectiveness of Brix 3000 and adjunctive PDT performed with the FotoSan 630 Intro Kit. **Methods**: This study included 30 children aged 4–7 years with carious lesions on primary molars classified as ICDAS II code 06. The lesions were allocated into two groups: Group 1 (the control group), encompassing 15 lesions excavated using Brix 3000 only, and Group 2 (the experimental group), encompassing 15 lesions excavated with Brix 3000 followed by adjunctive PDT with the FotoSan 630 Intro Kit. A total of 75 microbiological samples were collected: 30 from infected dentin before excavation, 30 from partially infected dentin after Brix 3000, and 15 (experimental group only) after subsequent PDT. **Results**: The results revealed a wide diversity of cariogenic microorganisms in the infected dentin, with S. mutans being the most frequently isolated and present in the highest quantities. Using Brix 3000 reduced microbial diversity and quantity following excavation of partially infected dentin, although *S. mutans* persisted at lower levels. **Conclusions**: After the additional photodynamic disinfection, no microorganisms were isolated from the partially infected dentin. Adjunctive PDT provides localized, non-invasive antimicrobial disinfection and can be integrated into minimally invasive caries management in primary teeth.

## 1. Introduction

Minimally invasive treatment is the leading approach in managing carious lesions in primary teeth, aiming to preserve as much of the hard dental tissue as possible [1,2]. In pediatric dentistry, this approach is particularly important due to the anatomical and morphological characteristics of primary teeth, the faster progression of carious lesions, and the need for gentle treatment methods that ensure patient comfort and clinical tolerance [3,4].

Within the context of minimally invasive dentistry, chemo-mechanical excavation is considered an alternative to conventional cavity preparation. Brix 3000 is a modern enzymatic agent used for the selective removal of infected dentin. It contains papain 3000 U/mg (10%), bio-encapsulated using advanced EBE technology (B.E.K. (buffered emulsion for encapsulation)), which stabilizes and immobilizes the enzyme while enhancing its activity [5].

Despite the advantages of chemo-mechanical excavation, microorganisms may persist in the remaining dentin. In this context, photodynamic therapy (PDT) is considered a promising adjunctive disinfection method [6]. PDT is an antimicrobial approach based on the interaction of a photosensitizer, light of a specific wavelength, and oxygen, resulting in the generation of reactive oxygen species, including singlet oxygen and short-lived free radicals. These reactive species induce rapid and irreversible damage to microbial cell structures, leading to cell death, while being rapidly deactivated, thereby minimizing the risk to surrounding healthy tissues. An important characteristic of photosensitizing agents is their preferential accumulation in damaged or pathogenic tissues, such as infected dentin, which enables targeted antimicrobial action [7,8]. Recent evidence also suggests that PDT may exert antimicrobial effects even under limited oxygen conditions, further supporting its potential clinical applicability [9].

The efficacy of PDT depends on the choice of photosensitizer, the light source and dose, and the exposure time. Clinically, red light (630–700 nm) is commonly used due to its tissue penetration properties. Among photosensitizers, methylene blue is widely applied for its photochemical activity, tissue penetration, and effectiveness against major cariogenic microorganisms, making it particularly suitable for photodynamic disinfection in carious lesion management [10].

Overall, the integration of PDT into minimally invasive pediatric dentistry represents an innovative application of biophotonic technology, providing a non-invasive, highly selective antimicrobial approach. This aligns with the emerging focus of this Special Issue on PDT, highlighting translational potential in both dental and broader medical contexts [11].

## 2. Results

### 2.1. Microbial Profile of Infected Dentin Prior to Excavation (Sample 1)

Table 1 presents the relative abundance of cariogenic microorganisms isolated from infected dentin (Sample 1) in Groups 1 and 2, before chemo-mechanical excavation with Brix 3000.

Before chemo-mechanical excavation with Brix 3000, infected dentin in both groups exhibited a diverse microbial composition dominated by cariogenic species. *Streptococcus mutans* and *Lactobacillus* spp. were the predominant microorganisms, confirming their central role in caries progression, while other oral streptococci and *Neisseria* spp. were present in lower proportions. The initial microbial profiles were comparable between the two groups, providing a consistent baseline for further analysis.

### 2.2. Microbial Profile of Partially Infected Dentin After Brix 3000 Excavation (Sample 2)

Table 2 shows the relative abundance of cariogenic microorganisms in partially infected dentin after Brix 3000 excavation in both groups.

After chemo-mechanical excavation with Brix 3000, the microbial load was reduced in both groups. Streptococcus mutans remained the predominant species, although at lower levels, highlighting its persistence within carious dentin. Other microorganisms were detected only sporadically and in low amounts. The presence of residual bacteria indicates that complete microbial elimination was not achieved in all cases, supporting the need for adjunctive antimicrobial disinfection. A shift toward lower CFU categories was observed in Sample 2 compared to Sample 1 in both groups following chemo-mechanical caries removal with Brix 3000.

### 2.3. Microbial Profile After Photodynamic Disinfection (Sample 3, Group 2)

Table 3 summarizes microbial results from partially infected dentin after additional disinfection with FotoSan in Group 2.

After PDT with FotoSan in Group 2, no microbial growth was detected in any sample. The uniform absence of bacterial growth across all post-PDT samples indicates the absence of detectable bacterial growth in all post-PDT samples following chemo-mechanical excavation with Brix 3000. These findings highlight the strong antimicrobial effect of PDT as an effective adjunctive disinfection procedure.

### 2.4. Representative Clinical Case

**Figure 1:**
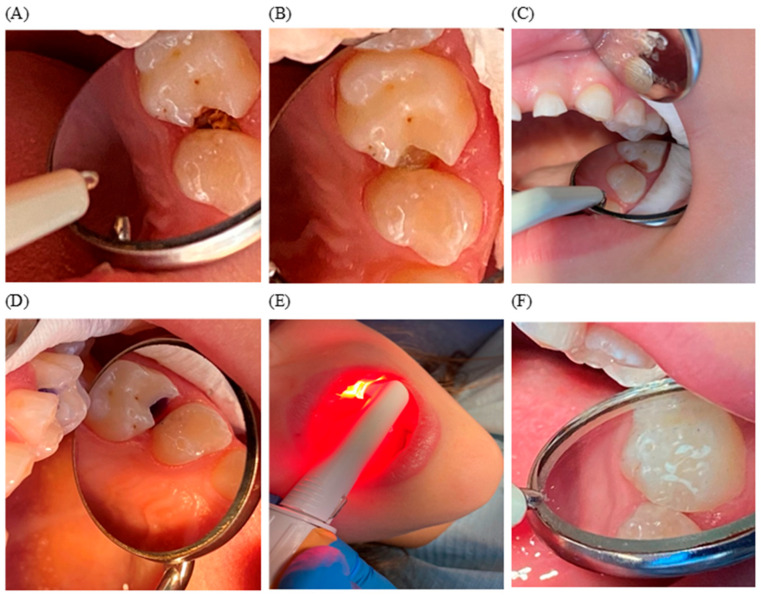
Sequence of Brix 3000 excavation and adjunctive FotoSan photodynamic disinfection in a 5-year-old patient. (**A**) Initial carious lesion exposure; (**B**) Application of Brix 3000 in the cavity; (**C**) Chemo-mechanical excavation with Brix 3000 to partially infected dentin (second microbiological sample collected); (**D**) Application of toluidine blue photosensitizer with syringe; (**E**) Activation of photosensitizer with FotoSan 630 Intro Kit using 4 mm blunt fiber (third microbiological sample collected); (**F**) Final restoration placement.

Figure 1 illustrates a 5-year-old patient treated with Brix 3000 excavation and subsequent photodynamic disinfection with FotoSan.

The clinical images in Figure 1 illustrate the sequential steps of chemo-mechanical excavation with Brix 3000, followed by adjunctive photodynamic disinfection with FotoSan in a 5-year-old patient. The images demonstrate the progression from initial carious lesion exposure (Figure 1A) to partial caries removal and subsequent PDT (Figure 1B–E), which resulted in the absence of detectable microbial growth after PDT, as confirmed by microbiological analysis. Final restoration placement is shown in Figure 1F.

## 3. Discussion

In the context of minimally invasive treatment of carious lesions, chemo-mechanical excavation with Brix 3000 has established itself as an effective method for the selective removal of infected dentin while preserving healthy tissues. Our results show a high diversity of microorganisms isolated from infected dentin in Sample 1 in both groups. *S. mutans* was the primary cariogenic microorganism, being the most abundant and isolated, reaching 1 × 10^5^–7 CFU in 13 cases per group. This confirms its leading role in the progression of the carious process and the high microbial activity in infected dentin. The second most frequently isolated microorganism at high concentrations was *Lactobacillus* spp.—73.3% in Group 1 and 66.7% in Group 2, reflecting the advancement and deepening of carious lesions. Other microorganisms, including *S. sanguis*, *S. parasanguis*, *S. mitis*, *S. epidermidis*, *Neisseria* spp., and *Actinomyces* spp., were isolated in lower quantities and only in individual cases.

After chemo-mechanical excavation with Brix 3000, microbial diversity significantly decreased. From partially infected dentin, *S. mutans* remained the most frequently isolated microorganism, albeit at lower levels, and was detected in all samples in both groups. This highlights the resilience of this microorganism even during the selective removal of infected dentin. All other microorganisms were detected in low quantities, and none were present in high amounts in partially infected dentin.

The presence of residual microorganisms necessitates additional antimicrobial measures. In our study, we applied PDT with FotoSan as an adjunctive disinfection procedure. Immediately after PDT, no microbial growth was observed in partially infected dentin, whereas reduced but still detectable microbial growth persisted after excavation with Brix 3000 in the control group. These results clearly demonstrate the high effectiveness of PDT as a gentle, localized antimicrobial method that does not damage healthy tissues or increase the risk of bacterial resistance [12,13,14,15]. The complete absence of microbial growth after PDT allows for successful one-visit indirect pulp capping during chemo-mechanical excavation with Brix 3000.

From a translational perspective, PDT can be regarded as a drug–device therapeutic system, in which a locally applied photosensitizer is activated by a dedicated light source to achieve targeted antimicrobial action. The most important characteristic of the interaction between biological tissues and photosensitizing agent molecules is their ability to selectively target only damaged cells or structures, such as infected dentin. PDT is based on the use of a photosensitive molecule which, after absorbing light of a specific wavelength, transitions into an excited state and induces a series of photochemical reactions leading to microbial inactivation. These processes occur locally, only within the irradiated area, which underlies the targeted nature of the antimicrobial effect [16,17].

The excited photosensitizer can participate in two main types of photochemical reactions—type I and type II—which occur simultaneously. In type I reactions, the triplet state of the photosensitizer interacts with nucleic acids, lipids, and proteins through electron or hydrogen atom transfer, resulting in the formation of radicals and reactive oxygen species, including hydrogen peroxide, hydroxyl radicals, and superoxide anions. In type II reactions, the energy of the excited photosensitizer is transferred to molecular oxygen, leading to the formation of singlet oxygen, which represents a key cytotoxic agent for microbial cells. In addition to these classical pathways, type III and type IV reactions have been described by Scherer et al., demonstrating that PDT may exert cytotoxic effects even under hypoxic or oxygen-depleted conditions. Type III reactions involve direct interactions of the excited photosensitizer with intracellular targets, while type IV reactions are associated with light-induced photochemical or conformational changes, such as photoisomerization, which facilitate binding to target biomolecules and enhance antimicrobial efficacy [18].

The effectiveness of PDT depends on several parameters, including the applied light dose, exposure time, and wavelength, which determines the depth of tissue penetration [9,19]. In clinical practice, photosensitizers are primarily activated by red light in the range of 630–700 nm, providing adequate penetration into demineralized dentin. In addition to various light sources, such as lasers and light-emitting diode systems, photosensitizers such as methylene blue are widely used. Due to its positive molecular charge and low molecular weight, methylene blue exhibits high efficacy against cariogenic microorganisms and enables the generation of significant amounts of singlet oxygen upon light irradiation [11,16,17].

Several clinical and in vivo studies have evaluated the antimicrobial effectiveness of PDT as an adjunct to selective caries removal, allowing direct comparison with the findings of the present study [20,21]. Numerous studies in the literature report a positive effect of PDT against residual microorganisms following excavation of carious dentin. Alves et al. studied 20 children with active carious lesions in primary molars and observed a statistically significant reduction in *S. mutans* after selective excavation and subsequent PDT, with a reduction of 76.4% after excavation and 92.6% after PDT [12]. Similar results have been reported by other authors, all concluding that PDT effectively disinfects residual dentin [22,23,24].

Additional studies compare different photosensitizers and light sources for *S. mutans* sterilization, showing that the combination of a photosensitizer and an appropriate wavelength significantly reduces microbial counts compared to their individual use [13,25]. Soria-Lozano et al. confirmed the high antimicrobial efficacy of PDT in the treatment of carious lesions in pediatric dentistry [26]. Azizi et al. demonstrated complete in vitro reduction in *S. mutans* using methylene blue and indocyanine green activated by lasers with wavelengths of 660 and 820 nm [13]. All these findings support the observations of the present study.

This study has several limitations that should be considered when interpreting the results. First, the relatively small sample size limits the generalizability of the findings and warrants cautious extrapolation to broader clinical populations. Second, microbiological data were analyzed using categorical CFU ranges, which restricted the application of more advanced quantitative statistical methods. In addition, the absence of detectable bacterial growth in post-PDT samples precluded inferential statistical analysis for this stage. Furthermore, the study was limited to a single PDT system and protocol, and long-term clinical outcomes were not assessed. Future studies with larger sample sizes, quantitative molecular techniques, and extended follow-up periods are needed to further validate the clinical effectiveness of PDT as an adjunctive disinfection method in minimally invasive pediatric dentistry.

## 4. Materials and Methods

### 4.1. Study Design and Ethical Considerations

This in vivo clinical study was conducted in accordance with the Declaration of Helsinki. Ethical approval was obtained from the Ethics Committee of the Medical University of Sofia (protocol code KENIMUS 05/20 February 2019). Written informed consent was obtained from the parents or legal guardians of all participating children prior to inclusion in the study.

### 4.2. Study Population and Inclusion Criteria

The clinical study included 30 children aged 4 to 7 years presenting with at least one carious lesion affecting a primary molar, classified as ICDAS II code 06. The included teeth exhibited deep carious lesions with vital pulp, without clinical symptoms of irreversible pulpitis, and were treated according to a protocol for selective caries removal and indirect pulp treatment.

All included teeth were clinically consistent with asymptomatic deep carious lesions in primary molars and showed no signs of acute symptomatology or irreversible pulp inflammation.

### 4.3. Group Allocation

The children were allocated into the following study groups:

- Group 1 (control group): 15 children treated with chemo-mechanical caries removal using Brix 3000 only.
- Group 2 (experimental group): 15 children treated with chemo-mechanical caries removal using Brix 3000, followed by an additional antimicrobial photodynamic disinfection procedure using the FotoSan630 Intro Kit.

### 4.4. Inclusion and Exclusion Criteria

The inclusion and exclusion criteria applied in this study are summarized in Table 4.

### 4.5. Clinical Procedures

#### 4.5.1. Chemo-Mechanical Caries Removal with Brix 3000—Group 1 (Control Group)

The carious lesion was accessed, and a microbiological sample was collected from the infected dentin prior to excavation (Sample 1). Brix 3000 (Brix S.R.L., Carcarañá, Argentina) was applied into the cavity in an approximate amount corresponding to the manufacturer’s recommended quantity, using a Koine sickle excavator (Koine Italia snc, Milan, Italy), and left in place for 2 min according to the manufacturer’s instructions.

The infected dentin was removed from the enamel–dentin junction and cavity walls until sound, non-carious dentin was achieved. Fluorescence control was performed using the ProFace W&H system (Bürmoos, Austria, W&H Dentalwerk Bürmoos GmbH) until the absence of fluorescence was observed. Excavation of the cavity floor was continued until partially infected (leathery) dentin was obtained, verified by ProFace fluorescence control showing red fluorescence with pale pink areas. A second microbiological sample was collected from the partially infected dentin (Sample 2).

A calcium hydroxide liner (caviLINE; Advanced Healthcare Ltd., London, UK) was applied, and the cavity was restored with a compomer restorative material.

#### 4.5.2. Chemo-Mechanical Caries Removal with Brix 3000 and PDT—Group 2 (Experimental Group)

The initial clinical and chemo-mechanical caries removal procedures in Group 2 were performed in the same manner as described for Group 1 (Section 4.5.1), including access to the carious lesion, application of Brix 3000, selective removal of infected dentin under fluorescence control, and collection of microbiological samples from infected dentin (Sample 1) and partially infected dentin (Sample 2).

Following chemo-mechanical excavation, PDT was applied as an adjunctive disinfection procedure prior to final restoration.

Subsequently, photodynamic disinfection was performed using the FotoSan 630 Intro Kit LED system (CMS Dental, A/S, Roslev, Denmark). A medium-viscosity toluidine blue photosensitizer (FotoSan Agent; CMS Dental, A/S, Roslev, Denmark) with a concentration of 0.1 mg/mL was applied into the cavity using a syringe. The photosensitizer was activated using red LED light with a wavelength of 630 nm. According to the manufacturer’s technical specifications, the FotoSan 630 system provides an irradiance in the range of approximately 2000–4000 mW/cm^2^ within a wavelength range of 620–640 nm; at the maximum specified irradiance, a 10 s irradiation corresponds to an energy density of 40 J/cm^2^, and due to the small irradiation area of the light guide (diameter 4 mm; ~0.126 cm^2^), the total delivered energy per cycle is approximately 5 J.

Irradiation was performed for 10 s using the FotoSan 630 Blunt light guide with a diameter of 4 mm, following the manufacturer’s validated clinical protocol. If direct contact between the light guide and the photosensitizer was not achieved, light activation was repeated for an additional 10 s.

After activation, the cavity was rinsed with sterile saline solution, and a third microbiological sample was collected following the disinfection procedure (Sample 3). A calcium hydroxide liner (caviLINE, VOCO, Cuxhaven, Germany) was applied. The final restoration was performed using a compomer material supplied in compules (Glasiosite, VOCO, Cuxhaven, Germany).

### 4.6. Fluorescence-Guided Assessment

All stages of chemo-mechanical excavation in both groups were controlled using fluorescence-based evaluation with the ProFace system (W&H Dentalwerk Bürmoos GmbH, Austria), operating at a wavelength of 405 nm. During examination, protective eyewear with a filter allowing wavelengths up to 500 nm was used.

For assessment of residual dentin fluorescence at the cavity floor, the cavity walls, cleaned to sound, non-carious dentin and exhibiting no fluorescence, were used as reference controls.

The degree of dentin destruction was evaluated using the visual–tactile criteria proposed by Bjørndal, in combination with the following fluorescence criteria [27]:

- Infected dentin: Intense red or dark red fluorescence covering the entire carious dentin area;
- Partially infected dentin: Pink fluorescence with localized, limited red areas in the peripulpal dentin zone;
- Affected dentin: Pale pink fluorescence localized to isolated areas of the cavity floor, with no fluorescence in the remaining cavity.

### 4.7. Microbiological Methods

A total of 75 microbiological samples were collected from all carious lesions, as follows:

- Thirty samples from infected dentin prior to excavation with Brix 3000 (Groups 1 and 2);
- Thirty samples from partially infected dentin after excavation with Brix 3000 (Groups 1 and 2);
- Fifteen samples collected after additional photodynamic disinfection with the FotoSan 630 Intro Kit (Group 2 only).

The collected material was placed in sterile Eppendorf tubes containing an appropriate transport medium provided by the microbiology laboratory and delivered within 3–4 h to the microbiology laboratory of the Department of Medical Microbiology, Medical University of Sofia. The transport medium was used to preserve microbial viability and prevent overgrowth during transport. All samples were processed immediately upon arrival at the laboratory. The same transport conditions and time interval were applied uniformly to all samples in both study groups.

Culturing was performed on blood agar, selective agar for lactobacilli, and brain–heart infusion broth. Cultures were initially incubated for 24 h, and plates showing no visible growth were further incubated for an additional 24 h (total incubation time of up to 48 h) at 36 °C in a CO_2_-enriched atmosphere, in accordance with routine clinical microbiology protocols, as this temperature falls within the optimal range (35–37 °C) for oral bacterial growth.

**Figure 2:**
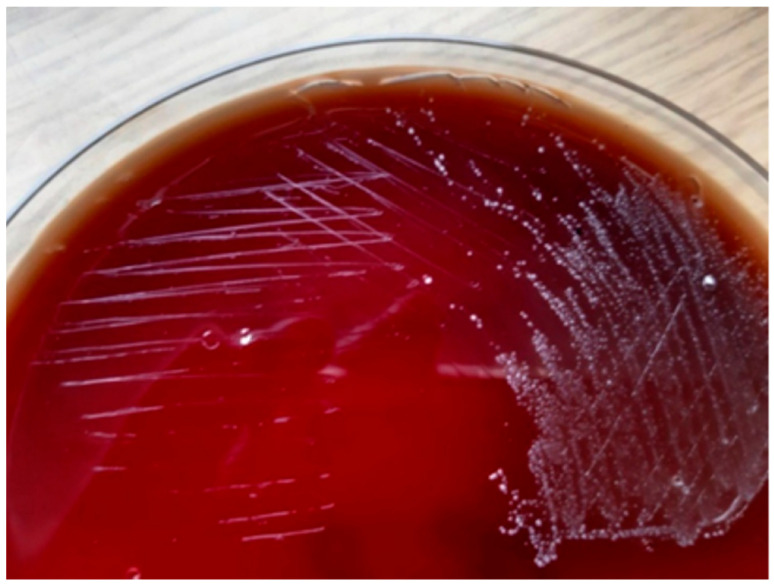
Streaking method used to inoculate the samples.

The streaking method used for sample inoculation is shown in Figure 2.

The streaking method was used to inoculate microbiological samples onto solid culture media in order to obtain isolated colonies. In cases of insufficient growth in primary solid media, the enriched broth cultures were subsequently subcultured onto additional agar plates. Pure cultures were isolated from morphologically suspicious colonies and further processed for microbiological identification.

**Figure 3:**
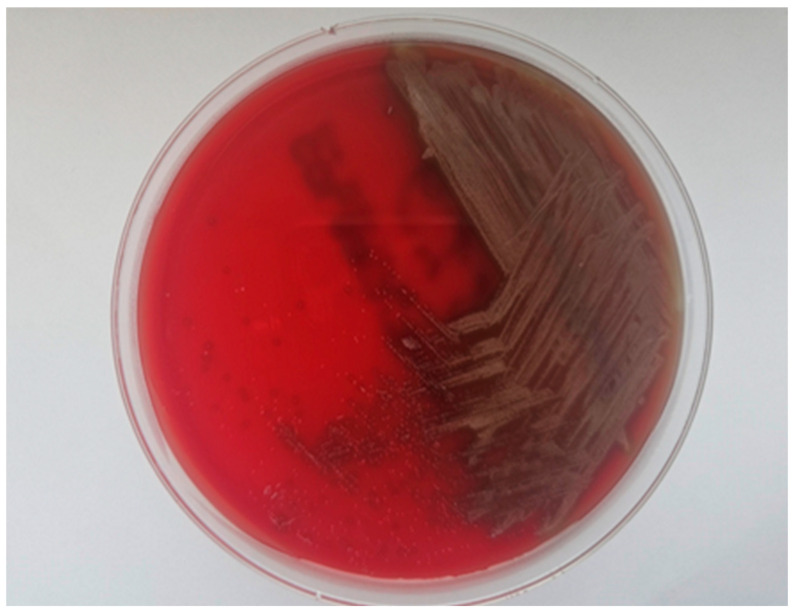
Growth of *Streptococcus mutans*, isolated from carious dentin, after overnight incubation.

The growth of *Streptococcus mutans* isolated from carious dentin is illustrated in Figure 3.

Microbial identification was performed based on colony morphology and standard microbiological identification procedures. Quantitative analysis was carried out by counting colony-forming units, and the results were expressed as colony-forming units per milliliter (CFU/mL).

### 4.8. Statistical Analysis

Microbiological data were analyzed using descriptive statistical methods and presented as absolute and relative frequencies (number and percentage). CFU ranges were treated as categorical variables. Comparisons between Sample 1 (infected dentin) and Sample 2 (partially infected dentin after Brix 3000) within each group were performed using Fisher’s exact test.

Post-PDT samples (Sample 3) were analyzed descriptively only, as no microbial growth was detected in any case, precluding the application of inferential statistical tests due to lack of variability.

## 5. Conclusions

In conclusion, the results of this study indicate that chemo-mechanical excavation with Brix 3000 markedly reduces microbial load in carious dentin, but residual microorganisms in partially infected dentin require additional disinfection strategies. The application of PDT with FotoSan results in the complete absence of microbial growth and highlights the high antimicrobial efficacy of the method. Based on these findings, PDT can be considered a safe, gentle, and promising adjunct to chemo-mechanical excavation with Brix 3000 in the treatment of carious lesions in primary teeth, ensuring maximal preservation of healthy tissues and effective control of cariogenic microorganisms.

## Figures and Tables

**Table 1:** Relative abundance of cariogenic microorganisms isolated from infected dentin (Sample 1) in Groups 1 and 2 before treatment with Brix 3000.

Cariogenic Microorganisms	Group 1: 1 × 10^5^–10^7^ CFU	Group 1: 1 × 10^3^–10^4^ CFU	Group 1: Without Growth	Group 2: 1 × 10^5^–10^7^ CFU	Group 2: 1 × 10^3^–10^4^ CFU	Group 2: Without Growth
*S. mutans*	13, 86.7%	2, 13.3%	0, 0%	13, 86.7%	2, 13.3%	0,
*S. sanguis*	3, 20%	2, 13.3%	10, 66.7%	1, 6.7%	3, 20%	11, 73.3%
*S. parasanguis*	2, 13.3%	2, 13.3%	11, 73.3%	2, 13.3%	3, 20%	10, 66.7%
*S. mitis*	4, 26.7%	0, -	11, 73.3%	2, 13.3%	1, 6.7%	12, 80%
*Staphylococcus epidermidis*	2, 13.3%	2, 13.3%	11, 73.3%	2, 13.3%	2, 13.3%	11, 73.3%
*Neisseria* spp.	2, 13.3%	0, -	13, 86.7%	0, -	1, 6.7%	14, 93.3%
*Actinomyces* spp.	1, 6.7%	3, 20%	11, 73.3%	0, -	3, 20%	12, 80%
*Lactobacillus* spp.	11, 73.3%	3, 20%	1, 6.7%	10, 66.7%	1, 6.7%	5, 33.3%

Note: Values are expressed as number of colonies (N) and corresponding percentage (%).

**Table 2:** Relative abundance of cariogenic microorganisms isolated from partially infected dentin (Sample 2) in Groups 1 and 2 after treatment with Brix 3000.

Cariogenic Microorganisms	Group 1: 1 × 10^5^–10^7^ CFU	Group 1: 1 × 10^3^–10^4^ CFU	Group 1: Without Growth	Group 2: 1 × 10^5^–10^7^ CFU	Group 2: 1 × 10^3^–10^4^ CFU	Group 2: Without Growth
*S. mutans*	-, -	13, 86.7%	2, 13.3%	-, -	13, 86.7%	2, 13.3%
*S. sanguis*	-, -	2, 13.3%	13, 86.7%	-, -	2, 13.3%	13, 86.7%
*S. parasanguis*	-, -	-, -	15, 100%	-, -	1, 6.7%	14, 93.3%
*S. mitis*	-, -	4, 26.7%	11, 73.3%	-, -	1, 6.7%	14, 93.3%
*Staphylococcus epidermidis*	-, -	2, 13.3%	13, 86.7%	-, -	3, 20%	12, 80%
*Neisseria* spp.	-, -	-, -	15, 100%	-, -	1, 6.7%	14, 93.3%
*Actinomyces* spp.	-, -	-, -	15, 100%	-, -	1, 6.7%	14, 93.3%
*Lactobacillus* spp.	-, -	-, -	15, 100%	-, -	1, 6.7%	14, 93.3%

Note: Values are expressed as number of colonies (N) and corresponding percentage (%).

**Table 3:** Relative abundance of cariogenic microorganisms isolated from partially infected dentin after additional photodynamic disinfection (Sample 3) in Group 2.

Cariogenic Microorganisms	Group 2: 1 × 10^5^–10^7^ CFU	Group 2: 1 × 10^3^–10^4^ CFU	Group 2: Without Growth
*S. mutans*	-, -	-, -	15, 100%
*S. sanguis*	-, -	-, -	15, 100%
*S. parasanguis*	-, -	-, -	15, 100%
*S. mitis*	-, -	-, -	15, 100%
*Staphylococcus epidermidis*	-, -	-, -	15, 100%
*Neisseria* spp.	-, -	-, -	15, 100%
*Actinomyces* spp.	-, -	-, -	15, 100%
*Lactobacillus* spp.	-, -	-, -	15, 100%

Note: Values are expressed as number of colonies (N) and corresponding percentage (%).

**Table 4:** Inclusion and exclusion criteria for study participation.

Inclusion Criteria	Exclusion Criteria
Children with written informed consent obtained from a parent or legal guardian	Children with negative behavior according to the Frankl behavior rating scale
Systemically healthy children	Presence of acute clinical symptoms at the time of examination
Children demonstrating positive behavior according to the Frankl behavior rating scale	Presence of periapical radiographic changes affecting the examined teeth
Absence of acute clinical symptoms at the time of examination	
Absence of concomitant systemic diseases	
Absence of periapical radiographic changes in the examined teeth	

## Data Availability

The original contributions presented in the study are included in the article. Further inquiries can be directed to the corresponding author.

## Abbreviations

The following abbreviations are used in this manuscript:

PDT	Photodynamic Therapy
ICDAS II	International Caries Detection and Assessment System II
CFU	Colony-Forming Unit
CFU/mL	Colony-Forming Units per Milliliter
LED	Light-Emitting Diode
B.E.K.	Buffered Emulsion for Encapsulation
EVE	Enzyme Vehicle Encapsulation
